# Decoding Brain Signals from Rapid-Event EEG for Visual Analysis Using Deep Learning

**DOI:** 10.3390/s24216965

**Published:** 2024-10-30

**Authors:** Madiha Rehman, Humaira Anwer, Helena Garay, Josep Alemany-Iturriaga, Isabel De la Torre Díez, Hafeez ur Rehman Siddiqui, Saleem Ullah

**Affiliations:** 1 Institute of Computer Science, Khwaja Fareed University of Engineering & Information Technology, Rahim Yar Khan 64200, Pakistan; humaira.anwer@kfueit.edu.pk (H.A.); hafeez@kfueit.edu.pk (H.u.R.S.); saleem.ullah@kfueit.edu.pk (S.U.); 2 Universidad Europea del Atlantico, Isabel Torres 21, 39011 Santander, Spain; helena.garay@uneatlantico.es; 3 Universidade Internacional do Cuanza, Cuito EN 250, Angola; 4 Universidad de La Romana, Edificio G&G, C/Héctor René Gil, Esquina C/Francisco Castillo Marquez, La Romana 22000, Dominican Republic; josep.alemany@uneatlantico.es; 5 Facultad de Ciencias Sociales y Humanidades, Universidad Europea del Atlántico, Isabel Torres 21, 39011 Santander, Spain; 6 Departamento de Ciencias de Lenguaje, Educación y Comunicaciones, Universidad Internacional Iberoamericana Arecibo, Arecibo, PR 00613, USA; 7 Department of Signal Theory, Communications and Telematics Engineering, University of Valladolid, 47011 Valladolid, Spain; itordie@gmail.com

**Keywords:** BCI, EEG, visual classification, rapid-event design, block design

## Abstract

The perception and recognition of objects around us empower environmental interaction. Harnessing the brain’s signals to achieve this objective has consistently posed difficulties. Researchers are exploring whether the poor accuracy in this field is a result of the design of the temporal stimulation (block versus rapid event) or the inherent complexity of electroencephalogram (EEG) signals. Decoding perceptive signal responses in subjects has become increasingly complex due to high noise levels and the complex nature of brain activities. EEG signals have high temporal resolution and are non-stationary signals, i.e., their mean and variance vary overtime. This study aims to develop a deep learning model for the decoding of subjects’ responses to rapid-event visual stimuli and highlights the major factors that contribute to low accuracy in the EEG visual classification task.The proposed multi-class, multi-channel model integrates feature fusion to handle complex, non-stationary signals. This model is applied to the largest publicly available EEG dataset for visual classification consisting of 40 object classes, with 1000 images in each class. Contemporary state-of-the-art studies in this area investigating a large number of object classes have achieved a maximum accuracy of 17.6%. In contrast, our approach, which integrates Multi-Class, Multi-Channel Feature Fusion (MCCFF), achieves a classification accuracy of 33.17% for 40 classes. These results demonstrate the potential of EEG signals in advancing EEG visual classification and offering potential for future applications in visual machine models.

## 1. Introduction

Electroencephalogram (EEG) imaging is a method used to assess the electrical activity of neurons in the brain. As the brain controls all bodily organs, brain signals change based on an individual’s mental state, cognitive processes, visual inputs, and other influencing elements [1,2]. It is well established that brain activity recordings contain specific information about visual object categories [3,4]. However, recognizing object classes in textual or video data is simpler than in brain signals, which is still a challenge for researchers [5,6]. Studies on EEG signal processing have identified the occipital lobe as the region of the brain responsible for visual perception, including the recognition of objects, as well as their shapes, colors, distances, and materials [7]. The occipital lobe can perform visuospatial processing and associated memory formation within a maximum of 200 ms [7,8]. Due to this biological connection, during EEG signal acquisition, visual stimuli are shown for 2 s, with 1 s breaks between consecutive stimuli. Research has shown that the rapid processing capabilities of the occipital lobe are crucial for accurate and timely visual perception [8]. This understanding has been pivotal in the development of EEG-based systems for object recognition [9].

Brain signals hold manifold information, reflecting a range of motor imagery tasks, emotional processes, sensory/auditory tasks, and cognitive behaviors [10,11]. This information can be utilized for a variety of endeavors, like for the recognition of emotions [12,13,14], sleep stages [15,16,17], prediction of critical thinking [18], speech activity detection in mute patients [19,20], etc. Various techniques are utilized for brain signals collection like fMRI (functional magnetic resonance imaging) [21,22], PET (positron emission tomography) [23], ECoG (electrocorticography) [24], MEG (magnetoencephalography) [25], and EEG [26]. FMRI and PET data provide great spatial resolution, but due to their lack of a temporal nature, cannot be used for visual object recognition [21,23]. The ECoG technique yields data with excellent temporal and spatial resolution but is highly invasive, as it requires the electrodes to be placed directly on the brain and not on the scalp [24]. MEG is used to measures the magnetic fields around the brain and is conducted inside a shielded room environment to avoid external electromagnetic noise. It provides high temporal and spatial resolutions and is a noninvasive technique [25]. However, due to the high cost and immobility of MEG, these devices are hard to use [27]. EEG also offers data with high temporal resolution that are well suited for object classification tasks [25].

EEG signals are classified into various frequency bands, like alpha (8–12 Hz), beta (13–25 Hz), theta (4–7 Hz), and gamma (30–80 Hz). Alpha and theta frequencies correspond to a person’s relaxed state with their eyes closed. Beta and gamma bands are known for recognizing critical thinking, problem solving, and visual recognition in the brain [28] and are mostly used in EEG visual recognition tasks [29,30,31,32].

The Temporal Stimulation Design (TSD) for signal acquisition greatly affects the EEG signal. Studies have shown that TSD is performed using a block design or a rapid-event design [33,34]. In the former, a person is continuously shown a block of images from the same class without any rest between images for the signal waveform to return to its baseline, distorting the next waveform [35,36]. In the latter design, the person is shown random images from different classes with an interval between each image. This interval is provided so that the excited neurons can reach the baseline so as not to interfere with the waveform of the next signal [37,38].

The block design in temporal stimulation is helpful in the detection of brain activity, such as epilepsy, neural activity in a brain part, tumors, and motor imagery signals [39,40,41]. The rapid-event design is well suited for classification tasks to distinguish one brain signal from another [33].

A possible reason behind the low accuracy in the visual classification task is that the EEG signals are non-stationary [35,42,43]. Their statistical features, such as mean and variance, change over time. A study by Miladinović et al. [44] indicated that the non-stationarity of EEG signals can cause shifts in feature covariance with time. To effectively capture and understand these signal-shifting dynamics, sophisticated analytical techniques are needed.

The rest of this paper is structured as follows: Section 2 provides an overview of related work, highlighting the datasets used in this task to date, as well as their usages and design techniques. In Section 3, we provide a detailed description of the dataset, as well as the data processing and feature extraction processes and the proposed classifiers. Section 4 presents the experimental results in detail. In Section 5, a detailed discussion about the experiments, the achieved results, and comparisons with state-of-the-art approaches is presented. Finally, Section 6 concludes the paper, summarizing key findings and discussing future research.

## 2. Related Work

In 2017, Spampinato et al. [34] published an article on the classification of visual objects through EEG signals using their self-made dataset. The authors reported an accuracy of 93.91%. Subsequently, the code and data were made publicly available, leading to numerous publications [33,34,45,46,47,48,49,50,51]. All of these studies incorporated the same data and achieved improvements in accuracy up to 97.13%.

In 2020, Hammad et al. [52] and Renli et al. [33] used the same dataset and code initially released in [34], and claimed that the achievement of high classification accuracy on this task is not due to the model architecture or the EEG signal but the following:

1. Usage of a block design during signal acquisition.
2. No preprocessing employed, i.e., usage of unfiltered data, resulting in training on noisy data.
3. Test data are sourced from the same block as the training data.

Hammad et al. [45,52], Renli et al. [33], and Hari [32] substantiated their points by the following means:

1. EEG data collected on a set of object classes/images identical to that utilized by Spampinato et al. [34];
2. Application of the same block design technique;
3. Adoption of similar preprocessing methods;
4. Utilization of test data sourced from the same block as the training data.

Following these steps, they achieved the highest classification accuracy in KNN, i.e., 100%. Other models like SVM, MLP, 1D CNN, and LSTM also performed very well, proving that a block design results in an accuracy boost in the object classification task. The authors concluded that in a block design, the rise in electrical potential in the brain is not given enough time to return to its baseline, contaminating the waveform of the next signal [33,45,52].

Hammad et al. [45,52] and Renli et al. [33] gathered data using a rapid-event temporal stimulation design with the same object classes as used before and performed prepossessing; the achieved results achieved astonishing, i.e., the accuracy degraded to 5.6%. Renli et al. [33] claimed that the data collected, used, and released in [34] suffer from irreparable contamination, i.e., all the image signals are contaminated by the next signal.

With the use of EEG signals in an event-related design rather than a block design, the accuracy of the signal is severely compromised. Researchers [29,31,53,54,55,56] have collected data for the EEG classification task using the rapid-event approach, varying the number of classes and incorporating different object classes. All of these studies [5,32,47,50,57,58,59] suggest that higher accuracy in EEG object classification is possible for only a low number of classes using rapid-event temporal stimulation. The authors of [45] published comments on [46] in Transactions on Pattern Analysis and Machine Learning, substantiating the point that using test data from the same block as the train data resulted in high accuracy in block design. Using the same data and code, only applying cross validation by leaving one block out in each turn, resulted in very low accuracy.

Various studies have been conducted to collect data using rapid-event and block designs on different object classes. A summary of EEG dataset collection efforts for EEG visual classification tasks are provided in Table 1. Datasets collected over time have used a variety in stimuli, temporal stimulation designs, numbers of classes, numbers of images per class, numbers of subjects, devices, numbers of channels, and sampling rates. Classification accuracies achieved on varying datasets is presented in Table 2.

A systematic summary of literature review considering the used dataset, applied ML models, and achieved accuracy is provided in Table 2. Higher accuracy trends can be seen in the data when a block design is used and when a lower number of classes is applied in a rapid-event design. A block design applied to 40 classes achieved a maximum of 100% accuracy, while a rapid-event design applied to the same classes but with a differing class image achieved maximum accuracy of 5.6%. During this extensive literature survey, we have identified the following several factors that cause low accuracy in the EEG visual classification task:

1. Determination of the optimal number of object classes to increase accuracy, as a low number of classes results in higher accuracy and vice-versa;
2. Lack of exploration of the use of a rapid-event design versus a block design during EEG signal acquisition [32,45,52,62];
3. Selection of channels, which contributes to accuracy boosts using channel selection techniques such as the linear removal of channel drops in accuracy to chance, as reported in [27,32].
4. Ensuring accurate labeling of data as incorrect or arbitrary labeling of events in block and rapid-event designs, resulting in accuracy boosts, reported in an analysis by Ren Li et al. [33] (page 318, Section 2 point e).
5. Implementation of effective preprocessing techniques to enhance data quality, as raw data result in higher accuracy than filtered data, as reported in [32,34,45,52].

## 3. Materials and Methods

**Figure 1:**
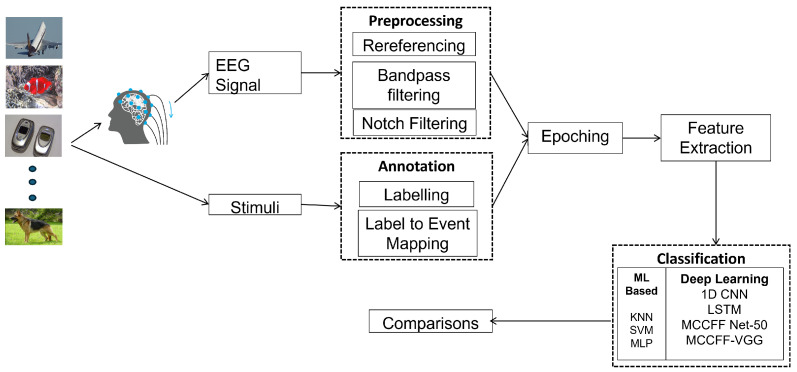
Diagram of the proposed methodology.

The proposed methodology, as presented in Figure 1, encompasses many essential steps in the processing and classification of EEG signals. First, the dataset is separated into EEG signals and annotations. Then, the signals are rereferenced to the mastoids to remove noise and artifacts. The signal is then bandpass-filtered to eliminate undesired frequencies. Subsequently, a notch-filtering technique is employed to eliminate any power-line interference. The EEG signals are divided into epochs, from which features are derived. Feature selection is a process that determines the most significant features for classification. This is accomplished using techniques like filters. Ultimately, classifiers are trained, then compared in order to evaluate their performance using metrics like accuracy and sensitivity. These metrics are crucial for the assessment and comparison of different classification methods.

### 3.1. Dataset Description

The dataset used in this study comprises publicly available data originally collected by [52]. In this research, we refer to it as ImageNet comprises a subset of images taken from the ILSVRC (ImageNet Large-Scale Visual Recognition Challenge) dataset. This is one of the largest EEG Signal datasets. Comprehensive details about the dataset are provided in Table 3. EEG signals were recorded while subjects viewed image stimuli from random object classes.

**Figure 2:**
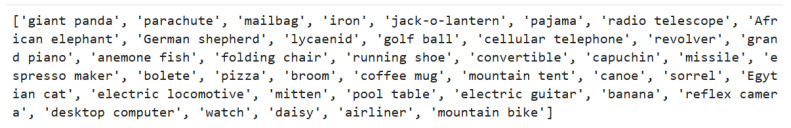
Classes used as visual stimulus.

**Figure 3:**
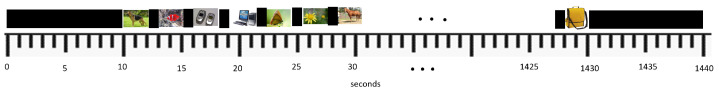
Timeline of the visual stimuli shown to subjects.

The dataset comprises of 40 classes with 1000 images each. The complete dataset is built on 40,000 images. Figure 2 shows the object classes used as visual stimuli. For each session, 10 images are randomly selected from each class, resulting in 100 sets of 400 images each. During each 1440 s session, the subject viewed 400 randomly ordered images. A total of 100 sessions were conducted, each approximately 1440 s in duration, employing a rapid-event temporal design. Figure 3 represents the baseline of these sessions. Each session begins and ends with 10 s of blank, followed by 2 s of visual stimulus and 1 s of a blank screen. The blank screen, displayed as a black screen, allows the subject’s brain signal to return to baseline before presenting the next image. This method ensures that the signal from each new stimulus does not interfere with the preceding signal.

### 3.2. Preprocessing and Feature Extraction

The raw EEG data from 99 brain data format (BDF) files were initially unprocessed. To manage computational resources effectively, the MNE library in Python was employed to read files in batches of two. Each file contained approximately 1440 s of EEG signals from 105 sensory positions, consisting of 104 channels and 1 stimulus channel providing event onset information. Separate event files were maintained using the same visual object sequence shown to the subjects, which was unique for each file/setting. The data were sampled at 4096 Hz, resulting in ((400 × 2 s + 1 s) × 4096) = 4,915,200 time points, with an additional (10 s + 10 s) × 4096 = 81,920 time points of start and end session blanks. The processing steps after reading the file include the following:

1. Raw EEG data are rereferenced to the mastoids to remove external noise and artifacts.
2. The data are bandpass-filtered by applying a zero-phase FIR filter from the MNE library. This filter eliminates phase shifts and gradually cuts off frequency components below 14 Hz and above 71 Hz, so no ringing artifacts remain in the signal.
3. A notch filter at 49–51 Hz is applied to remove any power-line noise.
4. The data are then epoched based on events, starting from −0.5 s and ending at 2.5 s for each event. The length of the epochs retrieved at this stage for a batch of files is 4,997,120 × 104. A total of 4,997,120 of the time points are considered rows, while 104 represent the sensory positions.
5. Events corresponding to 400 visual stimuli are extracted from the stimulus channel and assigned unique class labels for all 40 classes.
6. The data are then annotated using the unique labels and epoch data.

After signal preprocessing, the subsequent crucial step is feature extraction. This step is particularly significant due to the inherent complexity of EEG signals, i.e., they are non-stationary, non-linear, and non-Gaussian [30,63]. Given the temporal nature of EEG signals, preserving their effectiveness requires a statistical feature analysis and extraction approach. EEG is recorded at a 4096 Hz sampling frequency. This constitutes a huge amount of data that cannot be fed directly into any model. Therefore, in order to group the data while preserving effectiveness, various statistical methods were evaluated to determine the most effective, such as the mean, standard deviation, and mean of absolute values of first and second differences [63,64] were applied. The best results were achieved by performing the standard deviation on each of the channels per epoch.

### 3.3. Proposed Classifiers

This study employed seven different classifier models, each with an architecture optimized for EEG data. The machine learning learning models include k nearest neighbor (KNN), support vector machine (SVM) and multilayer perceptron (MLP). Deep learning models used in this study include are 1D Convolutional Neural Networks (1D CNNs), Long Short-Term Memory (LSTM), and the proposed MCCFF with ResNet and VGG models. We only adopted the ResNet-50 and VGG architectures, i.e., the models were trained on the dataset from scratch. The input shape to the deep learning models is a 3D matrix consisting of events, channels, and features. Owing to their long-term dependencies the 1D CNN and LSTM architectures are excellent choices for time-series data. The parameters for all these models are presented in Table 4.

The best results with the KNN classifier were achieved with a k value of 5. The SVM model used a polynomial kernel with a C value of 20 and gamma set to 1. The MLP classifier was configured with 1500 hidden layers and a maximum of 2000 iterations. For the 1D CNN model, tuning the learning rate and epochs and incorporating a batch normalization layer with dropout layers significantly improved performance and lowered the chance of overfitting, respectively. The LSTM model adopted a similar approach to the 1D CNN, with a learning rate of 0.005 sparse categorical cross-entropy. It employed a batch size of 100 and 150 epochs, using batch normalization with an Adam optimizer. The number of epochs and batch normalization were crucial factors in enhancing accuracy. The architectures of the 1D CNN and LSTM are presented in Table 5.

**Figure 4:**
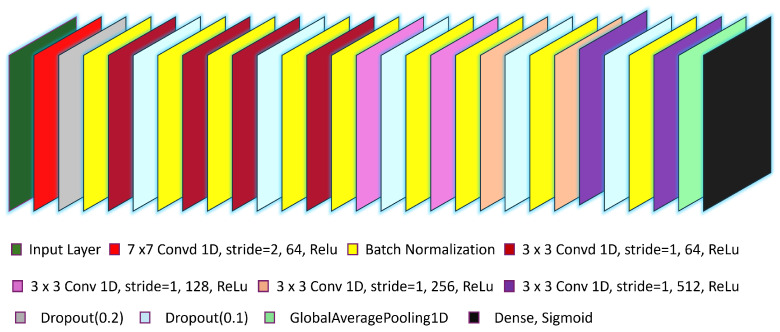
Proposed MCCFF model architecture based on ResNet-50.

This study proposes a robust MCCFF model based on the ResNet-50 architecture. The MCCFF ResNet model is capable of handling 40 object classes, each with 1000 images.The model utilizes sets of EEG images as input and employs encoders to extract features in the time domain. The model architecture includes an initial layer of 1D convolution with a kernel size of 7 × 7, a stride of 2, and 64 filters, using ReLU as the activation function, followed by batch normalization and a dropout. The fourth and fifth layers use 1D convolutions with a kernel size of 3 × 3, a stride of 1, and 64 filters each. Batch normalization and dropout are applied between layers. The following layers use the same convolution with 128 filters, and subsequent layers increase to 256 and 512 filters each. The architecture concludes with a global pooling layer and a fully connected layer. The detailed architecture is diagrammatically explained in Figure 4.

**Figure 5:**
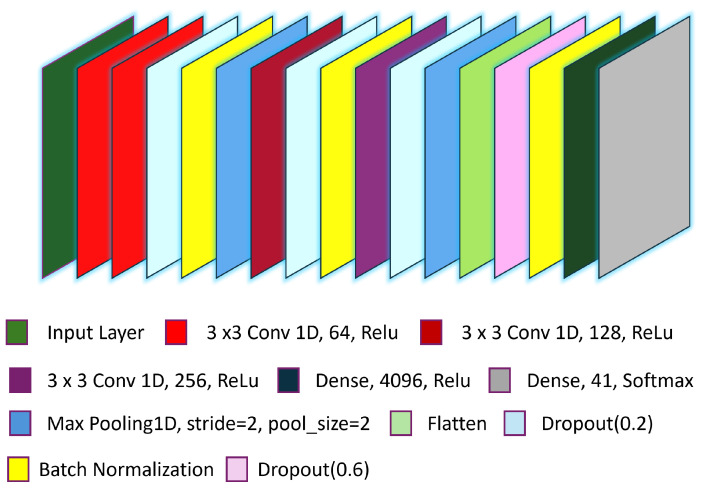
Proposed MCCFF model architecture based on VGG.

The proposed MCCFF VGG model is structured into four blocks of layers. In the first block, there are two layers of 3 × 3 1D convolutions with 64 filters each, followed by dropout, batch normalization, and a max pooling layer with a pool size of 2 and a stride of 2. The second block includes one layers of 3 × 3 1D convolution with 128 filters, followed by dropout, batch normalization, and a max pooling layer with the same pool size and stride. The third block consists of one layers of 3 × 3 1D convolution with 256 filters, followed by dropout and a max pooling layer with a pool size of 2 and a stride of 2. Each of these blocks uses the ReLU activation function. The final block contains two fully connected layers, each with 4096 dense units and dropout. The detailed architecture is diagrammatically explained in Figure 5.

## 4. Experimental Results

This section presents the results of the classification performed in this study. A detailed examination of the dataset was conducted using two different approaches across all seven models, using EEG data without filtering and with filtering. In this study, the models were rigorously validated using a 5-fold cross-validation technique. This technique partitions the dataset into five subsets, where each subset serves as a validation set once, while the remaining subsets are used for training. By rotating through all subsets for validation, this approach provides a comprehensive evaluation of each model’s generalizability and effectiveness. This methodological rigor enhances the study’s confidence in the reported classification accuracies and ensures that the results are robust and statistically sound.

### 4.1. Results with a No-Filtering Approach

The complexity of EEG signals necessitates caution in preprocessing, as human-defined methods can potentially degrade signal performance [65,66]. Prior research has shown that achieving high performance on rapid-design EEG is only possible for a low number of classes [5]. In the no-filtering approach, we utilized the complete dataset only by excluding point 2, i.e., bandpass filtering from Section 3.2. All other preprocessing steps were incorporated. The results achieved using this approach are shown in Table 6. It is evident that the proposed MCCFF Net-50 and MCCFF VGG models outperformed the others in terms of the maximum number of channels and window size. However, accuracy degraded to chance with the traditional models, which validates the work reported in [52].

### 4.2. Results with Filtered Data

The data were filtered using the steps outlined in Section 3.2. Table 7 summarizes the averaged results across various classifiers, revealing distinct performance patterns. Traditional models such as KNN, SVM, and MLP achieved relatively lower precision, recall, F1 scores, and accuracies, ranging from approximately 0.4% to 5.59%. In contrast, advanced neural network models like LSTM and 1D CNN exhibited improved performance, with LSTM achieving a precision of 47.96%, recall of 8.25%, F1 score of 5.86%, and accuracy of 8.25%, while 1D CNN demonstrated a precision of 52.42%, recall of 13.0%, F1 score of 11.07%, and accuracy of 12.99%. MCCFF Net-50 achieved a precision of 44.34%, recall of 13.5%, F1 score of 8.73%, and accuracy of 13.50%, while MCCFF VGG achieved a precision of 54.47%, recall of 14.57%, F1 score of 4.31%, and accuracy of 14.57%.

## 5. Discussion

### 5.1. Effect of the Sensor Selection Strategy

**Figure 6:**
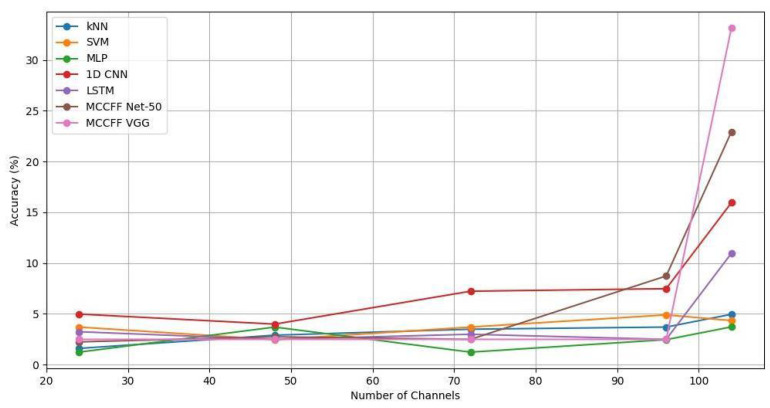
Accuracies of all the models for a 2500 ms time window and varying numbers of channels established on non-filtered data.

**Figure 7:**
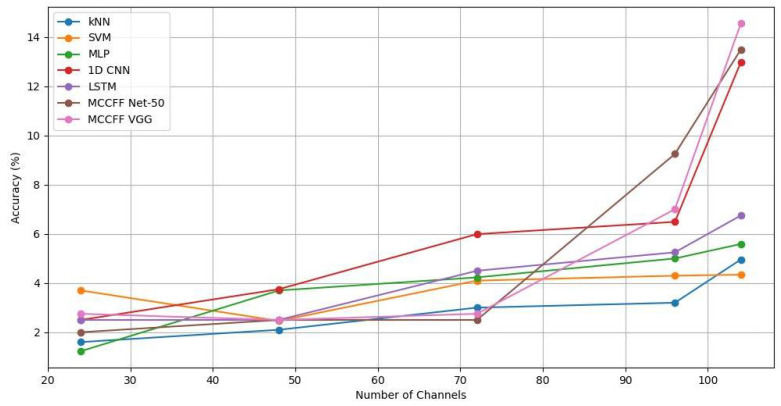
Accuracies of all the models for a 2500 ms time window and varying numbers of channels established on filtered data.

The bio-semi device used for signal acquisition uses four main sensory channels (A, B, C, and EXG), each equipped with 32 channels, except for EXG, which has 8. We used sequential feature selection (SFS) based on a backward elimination approach for channel selection [27]. Backward elimination eliminates the channels from the backward direction. The first elimination removes the sensory EXG group with channels. In the subsequent eliminations, eight channels were removed from each sensory group (A, B, and C), yielding 72, 48, and 24 channel configurations. In this way, SFS provided comprehensive coverage across all areas of the brain. This selection approach yielded significant results, with higher accuracies for 104 and 96 channels, whereas accuracy dropped notably with 72, 48, and 24 channels. The detailed results are provided in the Supplementary Materials. The accuracy trends of the 1D CNN, LSTM, and MCCFF models across a range of channels are shown in Figure 6 and Figure 7 for unfiltered and filtered data, respectively. Higher numbers of channel configurations yielded superior accuracies across models, with MCCFF VGG achieving accuracies of 14.57% and 7.0% on 104 and 96 channels, respectively, compared to lower accuracies on fewer channels (e.g., 2.75% and 2.5% for MCCFF Net-50 and MCCFF VGG, respectively on 72, 48, and 24 channels). These visualizations provide insights into the various models’ behaviors with varying numbers of channels, reinforcing the robustness of the MCCFF architectures in handling the complex temporal characteristics of EEG data.

### 5.2. Effect of Window Sizes on Signal Accuracy

**Figure 8:**
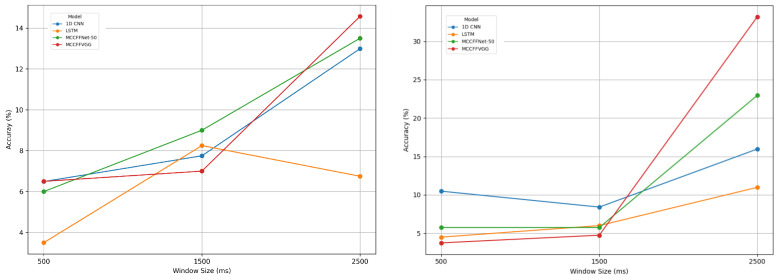
Effects of varying window sizes on filtered data (**Left**) and non-filtered data (**right**).

Figure 8 elucidates how varying window sizes influenced classifier performance. Longer window sizes resulted in higher accuracy and vice-versa. The analysis encompassed time-window slices of 2500 ms, 1500 ms, and 500 ms around events. Previous studies have used 1500 ms time windows, starting at 0s from the stimulus onset. In this study, we used time slices of −0.5 s before stimulus onset and 2.5 s, 1.5 s, and 0.5 s after stimulus onset to demonstrate the effects of longer and shorter window sizes on the data. The usage of −0.5 s before stimulus onset helps in capturing the baseline neural activity, thereby completing the signal waveform. This significantly improved the performance of the models. When shorter window sizes of 1.5 s and 0.5 s were used, crucial information needed for accurate recognition was missed, leading to the observed drop in performance for all models shown in Figure 8.

### 5.3. Comparison

A comparative study of the state of the art with the proposed method is provided in Table 8. Hari M Bharadwaj [32] achieved an accuracy of 17.6% on the same dataset using EEGNet. The experimental results suggest that the accuracies of the models (k-NN, SVM, and MLP) investigated in this study are in accordance with those reported in [32,52]. However, the proposed models following our multi-class, multi-channel feature fusion technique significantly improved the accuracies by up to 33.17%.

## 6. Conclusions

The field of EEG signal classification has seen significant advancements with the introduction of novel machine learning and deep learning approaches. However, due to the inherent complexity and non-stationary nature of EEG signals, achieving high classification accuracy remains challenging. Traditional models often struggle with the temporal and non-linear characteristics of these signals, necessitating the development of more sophisticated methods. The current study addresses these challenges by introducing the MCCFF-NET 50 and MCCFF-VGG models, which demonstrated substantial improvements in classification accuracy. The experimental results clearly show that these proposed approaches significantly outperform traditional models such as K-NN, SVM, and MLP, establishing a new benchmark in the field of object classification using visual EEG signals. While traditional models showed consistent but lower accuracies, the architectural enhancements in LSTM and 1D CNN led to notable performance improvements. Furthermore, this study revealed that the judicious selection of channels, covering the entire brain, significantly impacted classification accuracy. Future work will focus on exploring more sophisticated deep learning architectures and further optimizing preprocessing techniques. Additionally, the investigation of real-time applications and the expansion of the dataset to include more diverse stimuli could provide deeper insights and broader applicability in neuroscientific research and clinical diagnostics. The integration of multimodal data and the leveraging of transfer learning techniques also hold promise for future advancements in the field.

## Figures and Tables

**Table 1:** Summary of datasets used for EEG visual classification.

Ref#	Name of Dataset	Journal and Year	Stimulus	TSD	No. of Classes	No. of Images/ Clips per Class	No. of Subjects	Device/No. of Channels	Sampling Rate
[52]	ImageNet D1	Journal 2021	Image	Rapid Event	40	1000	01	BioSemi ActiveTwo recorder 104	4096 Hz
[34]	ImageNet D2	Journal 2017	Image	Block Design	40	50	06	ActiCap 128	1000 Hz
[33]	ImageNet D3	Journal 2021	Image	Rapid Event	40	50	6	BioSemi ActiveTwo recorder 104	4096 Hz
[33]	ImageNet V1	Journal 2021	Video	Rapid Event	12	32	06	BioSemi ActiveTwo recorder 104	4096 Hz
[53]	Stanford Dataset D4	Journal 2015	Image	Rapid Event	6	12	10	EGI HCGSN 128	1000 Hz
[29]	MPI DB D5	Journal 2010	Image	Rapid Event	3	4	04	ActiCap System 64	500 Hz
[54]	Things D6	Journal 2022	Image	Rapid Event	1854	10	10	Easy Cap 64	1000 Hz
[31]	ImageNet D7	Journal 2023	Image	Rapid Event	4	10	04	ActiCHamp 32	1000 Hz
[55]	MNIST D8	Journal 2024	Image	Rapid Event	11	116	01	Emotiv EPOC 14	128 Hz
[56]	Human dataset D9	Journal 2017	Image	Rapid Event	5	12	16	Easycap 74	1000 Hz
[60]	EEG-ImageNet D10	Journal 2024	Image (coarse-grained)	Block Design	40	50	16	-	1000 Hz
[60]	EEG-ImageNet D11	Journal 2024	Image (fine-grained)	Block Design	40	50	16	-	1000 Hz

**Table 2:** Summary of all the relevant literature.

Ref #	Year	Type	Dataset Utilized	Classes	TSD	Classifier	Accuracy
[60]	2024	Journal	EEG-ImageNet D11	40	Block Design	SVM	77.84%
						MLP	81.63%
						EEGNet	36.45%
						RGNN	70.57%
[60]	2024	Journal	EEG-ImageNet D10	40	Block Design	SVM	50.57%
						MLP	53.39%
						EEGNet	30.30%
						RGNN	47.03%
[61]	2024	Journal	ImageNet D1	40	Rapid Event	EEGVis_CMR (from EEG to Image)	17.9%
[57]	2024	Journal	MNIST D8	11	Rapid Event	RieManiSpectraNet	55%
[58]	2024	Journal	Human dataset D9	05	Rapid Event	LDA	68.75%
[59]	2023	Journal	Stanford Dataset D4	06	Rapid Event	LSTM	55.55%
						SVM	66.67%
[31]	2023	Journal	ImageNet D7	04	Rapid Event	SVM	36.22%
						CNN	64.49%
						LSTM-CNN	65.26%
						EEGNet	79.29%
[54]	2022	Journal	Things D6	1854	Rapid Event	AlexNet	15.4%
						ResNet-50	16.25%
						CORnet	21.05%
						MoCo	12.40%
[50]	2022	Journal	ImageNet D2	40	Block Design	SVM	82.70%
						RNN-based Model	84.00%
						Siamese Network	93.70%
						Bi-LSTM	92.59%
						HDRS-STF	99.78%
						BiLISTM+AttGW	99.50%
[5]	2023	Journal	Stanford Dataset D4	06	Rapid Event	LDA	40.52%
						ShallowConvNet	46.51%
						EENet	43.83%
						LSTM	38.06%
						EEG-Conv Transformer	52.33%
						TSCNN	54.28%
			Max Plank Institute Dataset [MPI DB]	03	Rapid Event	LDA	76.11%
						ShallowConvNet	77.42%
						EEGNet	77.79%
						LSTM	60.61%
						TSCNN	84.40%
[32]	2023	Journal	ImageNet D1	40	Rapid Event	LSTM	2.3%
						k-NN	2.1%
						SVM	3.0%
						MLP	2.8%
						1D CNN	2.4%
						EEGNet	17.6%
						SyncNet	3.7%
[45]	2022	Journal	ImageNet D2	40	Block Design	LSTM	2.7%
						k-NN	3.6%
						SVM	3.0%
						MLP	3.7%
						1D CNN	3.3%
						EEGNet	2.5%
						SyncNet	3.8%
						EEGChannelNet	2.6%
[33]	2021	Journal	ImageNet D3	40	Rapid Event	LSTM	2.9%
						k-NN	3.2%
						SVM	3.0%
						MLP	3.7%
						1D CNN	3.3%
[52]	2021	Conference	ImageNet D1	40	Rapid Event	1D CNN	5.1%
						LSTM	2.2%
						SVM	5.0%
						k-NN	2.1%
[62]	2019	Journal	ImageNet D2	40	Block Design	LSTM	63.1%
						k-NN	100%
						SVM	100%
						MLP	21.9%
						1D CNN	85.9%
			ImageNet D3	40	Rapid Event	LSTM	0.7%
						k-NN	1.4%
						SVM	2.7%
						MLP	1.5%
						1D CNN	2.1%
[46]	2020	Journal	ImageNet D2	40	Block Design	Inception v3 (from signals to images)	94.4%
[47]	2019	Journal	ImageNet D2	40	Block Design	Proposed LSTM-B	97.13%
[48]	2018	Conference	ImageNet D2	40	Block Design	Proposed Bidirectional LSTMs	97.3%
[49]	2018	Conference	ImageNet D2	40	Block Design	Proposed Region-level bi-directional LSTM	97.1%
[34]	2017	Conference	ImageNet D2	40	Block Design	GoogleNet	92.6%
						VGG	80.0%
						Proposed Method	89.7%

**Table 3:** Image-Net EEG data collection.

Device	BioSemi ActiveTwo recorder
Number of Subjects	1
Visual Stimuli	ILSVRC-2021
Total Classes	40
Images per Class	1000
Duration of Visual Stimuli	2 s with 1 s blanking
Sampling Frequency	4096 Hz
Data Resolution	24 bits
Temporal Stimulation Design	Rapid Event design

**Table 4:** Parameters of the classifiers used for EEG data after feature extraction.

Classifier	Parameters
KNN	k = 5
SVM	kernel = ‘poly’, C = 20, random_state = 1,gamma = 1, probability = True, class_weight = ‘balanced’
MLP	Hidden_layers = 1500, Max_iterations = 2000, random_state = 42
1D CNN	Learning rate = 0.0005, batch size = 100, epochs = 200, optimizer = Adam, loss = sparse_categorical_crossentropy, metrics = Accuracy, no of Layers = 15, activation = Relu, Softmax
LSTM	Learning rate = 0.005, batch size = 100, epochs = 200, optimizer = Adam, loss = sparse_categorical_crossentropy, metrics = Accuracy, no of Layers = 14, activation = Relu, Softmax
MCCFF Net-50	Learning rate = 0.005, Batch size = 120, epochs = 150, optimizer = Adam, loss = sparse_categorical_crossentropy, metrics = Accuracy, no of layers = 51, activation = Relu, Sigmoid
MCCFF VGG	Learning rate = 0.001, Batch size = 100, epochs = 200, optimizer = Adam, loss = sparse_categorical_crossentropy, metrics = Accuracy, no of layers = 16, activation = Relu, Sigmoid

**Table 5:** 1D CNN and LSTM model architectures.

1D CNN Model			LSTM Model		
**Layer**	**Neural Units/Kernel Size**	**Activation**	**Layer**	**Neural Units**	**Activation**
Conv1D	8 (3, 3)	ReLU	LSTM	8	ReLU
Dropout	0.1	-	Batch Normalization	-	-
Batch Normalization	-	-	MaxPooling1D	-	-
MaxPooling1D	(4, 4)	-	LSTM	16	ReLU
Conv1D	16 (3, 3)	ReLU	Dropout	0.2	-
Dropout	0.2	-	Batch Normalization	-	-
Batch Normalization	-	-	MaxPooling1D	-	-
MaxPooling1D	(4, 4)	-	LSTM	32	ReLU
Conv1D	32 (3, 3)	ReLU	Dropout	0.4	-
Batch Normalization	-	-	Batch Normalization	-	-
Flatten	-	-	MaxPooling1D	-	-
Dense Layer	16	ReLU	Flatten	-	-
Dropout	0.4	-	Dense Layer	32	ReLU
Batch Normalization	-	-	Dense (Output Layer)	41	Softmax
Dense (Output Layer)	41	Softmax			

**Table 6:** Results on non-filtered data. All results were validated using 5-fold cross validation leaving one fold out.

Classifier	Precision (%)	Recall (%)	F1 Score (%)	Accuracy (%)
KNN	2.49	4.96	2.82	4.96
SVM	7.51	4.34	3.93	4.34
MLP	14.31	3.72	2.85	3.72
Proposed Models				
LSTM	21.86	10.97	9.32	10.97
1d-CNN	47.369	15.96	13.591	15.960
MCCFF Net-50	48.11	22.94	20.53	22.94
MCCFF VGG	62.05	33.16	34.59	33.17

**Table 7:** Results achieved on filtered data. All the results were validated using 5-fold cross validation.

Classifier	Precision (%)	Recall (%)	F1 Score (%)	Accuracy (%)
KNN	40.0	4.9	3.4	4.96
SVM	5.1	4.34	4.1	4.34
MLP	13.5	5.59	6.51	5.59
Proposed Models				
LSTM	47.96	8.25	5.86	8.25
1d-CNN	52.42	13.0	11.07	12.99
MCCFF Net-50	44.34	13.5	8.73	13.50
MCCFF VGG	54.47	14.57	4.31	14.57

**Table 8:** Comparative analysis of the state of the art and the proposed methodology.

Study	Accuracy (%)
Hamad Ahmed et al. [52]	
1DCNN	5.1%
Hari M. Bharadwaj et al. [32]	
EEGNet	17.6%
Proposed Method	
MCCFF Net-50	22.94%
MCCFF VGG	33.17%

## Data Availability

The dataset of EEG signals used in this study for visual classification of objects with a rapid-event temporal design is available at https://ieee-dataport.org/open-access/dataset-object-classification-randomized-eeg-trials (accessed on 27 October 2024).

## Supplementary Materials

The following supporting information can be downloaded at: https://www.mdpi.com/article/10.3390/s1010000/s1.
