# Supplementary material for: Decoding Brain Signals from Rapid-Event EEG for Visual Analysis Using Deep Learning
Source: Sensors (Basel). 2024 Oct 30;24(21):6965. doi: 10.3390/s24216965 (PMC11548637; doi:10.3390/s24216965)
Supplement: Supplementary file 1 [file sensors-24-06965-s001.zip › sensors-3180536-supplementary.pdf]

1. Supplementary Material

The results achieved on varying the number of channels and window sizes on filtered and non filtered data is presented here in Table S1 and S2.

Table S1. Results achieved by varying the number of channels and window sizes on non-filtered data.

No of Channels	Window Size	KNN	SVM	MLP	1D CNN	LSTM	MCCFF Net-50	MCCFF VGG
104		4.96%	4.34%	3.72%	15.960%	10.97%	22.94%	33.17%
96		3.70%	4.9%	2.46%	7.48%	2.49%	8.73%	2.49%
72	2500ms	3.49%	3.70%	1.23%	7.23%	2.99%	2.49%	2.49%
48		2.9%	2.46%	3.70%	3.99%	2.49%	2.74%	2.49%
24		1.6%	3.7%	1.23%	4.98%	3.24%	2.24%	2.49%
104		2.25%	4.34%	2.484%	8.4%	5.99%	5.74%	4.74%
96		2.74%	3.1%	1.86%	7.5%	2.49%	4.24%	2.49%
72	1500ms	3.105%	1.24%	2.48%	5.48%	2.49%	5.99%	2.49%
48		2.74%	2.24%	2.0%	6.73%	3.74%	2.74%	2.49%
24		2.7%	1.24%	1.87%	3.74%	2.24%	2.74%	2.49%
104		1.86%	3.10%	2.48%	10.47%	4.49%	5.74%	3.74%
96		2.0%	1.24%	1.25%	6.73%	2.49	2.74%	2.49%
72	500ms	1.2%	1.20%	1.25%	8.7%	2.74	2.74%	2.49%
48		1.12%	3.6%	2.6%	6.23%	2.74	3.96%	2.49%
24		0.4%	2.45%	3.6%	4.98%	3.24	3.29%	2.49%

Table S2. Results achieved by varying the number of channels and window sizes on Filtered data.

No of Channels	Window Size	KNN	SVM	MLP	1D CNN	LSTM	MCCFF Net-50	MCCFF VGG
104		4.96%	4.34%	5.59%	12.99%	6.75%	13.50%	14.57%
96		3.2%	4.30%	5.0%	6.49%	5.25%	9.25%	7.00%
72	2500ms	3.0%	4.10%	4.23%	5.99%	4.50%	2.5%	2.75%
48		2.1%	2.46%	3.70%	3.75%	2.50%	2.5%	2.5%
24		1.6%	3.7%	1.23%	2.5%	2.5%	2.0%	2.75%
104		4.96%	4.34%	5.59%	7.75%	8.25%	9.0%	7.0%
96		4.96%	4.30%	5.46%	6.75%	4.50%	5.0%	5.0%
72	1500ms	4.40%	4.70%	4.23%	5.99%	3.75%	3.0%	2.75%
48		4.1%	2.46%	3.70%	4.749%	3.50%	4.25%	3.25%
24		1.6%	3.7%	1.23%	3.99%	4.25%	2.75%	4.0%
104		1.86%	3.10%	2.48%	6.49%	3.50%	6.0%	5.58%
96		2.0%	3.6%	2.67%	6.34%	2.50%	8.5%	6.5%
72	500ms	1.2%	4.6%	3.6%	5.49%	2.5%	4.0%	2.75%
48		1.12%	3.6%	2.6%	3.24%	2.5%	2.0%	3.75%
24		0.4%	2.45%	3.6%	2.5%	2.5%	2.5%	3.0%
